# Computed Tomography Radiomics Kinetics as Early Imaging Correlates of Osteoradionecrosis in Oropharyngeal Cancer Patients

**DOI:** 10.3389/frai.2021.618469

**Published:** 2021-04-09

**Authors:** Souptik Barua, Hesham Elhalawani, Stefania Volpe, Karine A. Al Feghali, Pei Yang, Sweet Ping Ng, Baher Elgohari, Robin C. Granberry, Dennis S. Mackin, G. Brandon Gunn, Katherine A. Hutcheson, Mark S. Chambers, Laurence E. Court, Abdallah S. R. Mohamed, Clifton D. Fuller, Stephen Y. Lai, Arvind Rao

**Affiliations:** ^1^Department of Electrical and Computer Engineering, Rice University, Houston, TX, United States; ^2^Department of Computational Medicine and Bioinformatics, University of Michigan, Ann Arbor, MI, United States; ^3^Department of Radiation Oncology, The University of Texas MD Anderson Cancer Center, Houston, TX, United States; ^4^Department of Radiation Oncology, European Institute of Oncology IRCSS, Milan, Italy; ^5^Department of Oncology and Hemato-Oncology, University of Milan, Milan, Italy; ^6^Department of Radiation Oncology, Peter MacCallum Cancer Centre, Melbourne, VIC, Australia; ^7^Department of Radiation Physics, The University of Texas MD Anderson Cancer Center, Houston, TX, United States; ^8^Department of Oncologic Dentistry and Prosthodontics, The University of Texas MD Anderson Cancer Center, Houston, TX, United States; ^9^Department of Head and Neck Surgery, The University of Texas MD Anderson Cancer Center, Houston, TX, United States; ^10^Department of Radiation Oncology, University of Michigan, Ann Arbor, MI, United States

**Keywords:** osteoradionecrosis, computed tomography, radiomics, longitudinal, radiotherapy, head and neck cancer, oropharyngeal cancer, functional principal component analysis

## Abstract

Osteoradionecrosis (ORN) is a major side-effect of radiation therapy in oropharyngeal cancer (OPC) patients. In this study, we demonstrate that early prediction of ORN is possible by analyzing the temporal evolution of mandibular subvolumes receiving radiation. For our analysis, we use computed tomography (CT) scans from 21 OPC patients treated with Intensity Modulated Radiation Therapy (IMRT) with subsequent radiographically-proven ≥ grade II ORN, at three different time points: pre-IMRT, 2-months, and 6-months post-IMRT. For each patient, radiomic features were extracted from a mandibular subvolume that developed ORN and a control subvolume that received the same dose but did not develop ORN. We used a Multivariate Functional Principal Component Analysis (MFPCA) approach to characterize the temporal trajectories of these features. The proposed MFPCA model performs the best at classifying ORN vs. Control subvolumes with an area under curve (AUC) = 0.74 [95% confidence interval (C.I.): 0.61–0.90], significantly outperforming existing approaches such as a pre-IMRT features model or a delta model based on changes at intermediate time points, i.e., at 2- and 6-month follow-up. This suggests that temporal trajectories of radiomics features derived from sequential pre- and post-RT CT scans can provide markers that are correlates of RT-induced mandibular injury, and consequently aid in earlier management of ORN.

## Introduction

Radiotherapy (RT) is a highly utilized modality in the treatment of head and neck (H&N) cancers with well-established local control and survival benefits (Pan et al., 2016). Advances in radiation delivery techniques from 2-dimensional (2D) and 3-dimensional (3D) techniques to intensity-modulated radiotherapy (IMRT) with the ability to manipulate the beam path to spare normal tissues has significantly improved cure rates and toxicity profile (Allison et al., 2014). Despite that, osteoradionecrosis is a late complication from radiation to the mandibular bone with a serious impact on the quality of life for a growing population of younger surviving head and neck cancer patients (Oh et al., 2004). The incidence of ORN varied between different modalities ranging from 2 to 40% in the conventional era to 0–6% in the IMRT era. Different risk factors were identified to play a role in the development of ORN following radiotherapy treatments (Allison et al., 2013; Zhang et al., 2017). Osteoradionecrosis has a great impact on the patients' quality of life if not detected and managed properly (Tucker et al., 2016; Wong et al., 2017). Diagnosis of ORN mainly relies on clinical and radiological tools such as computed tomography (CT) and magnetic resonance imaging (MRI) with their limited capacity for early detection (Tsien et al., 2014).

Fortunately, the recent advances in biomedical imaging were coupled with the rise of radiomics in terms of extracting quantifiable imaging features, possibly of high information yield and subsequent computation of these features kinetics (e.g., delta-radiomics) derived from sequential images (Cacicedo et al., 2016). Paired with machine learning techniques, we hypothesize that radiomic feature kinetics can characterize and distinguish mandibular bone subvolumes at higher risk of developing future ORN. These “temporal virtual digital biopsies” might have the potential to empower earlier intervention and hence improve patients' quality of life.

Consequently, the aims of this study are to:

1. Determine bone radiomic features derived from contrast-enhanced CT (CECT) images that are significantly different between ORN and non-ORN mandibular subvolumes.
2. Develop a predictive radiomic-based signature of ORN based on CECT temporal changes in high-risk mandibular subvolumes
3. Hypothesis generation for future prospective studies.

## Materials and Methods

### Study Population

**Figure 1:**
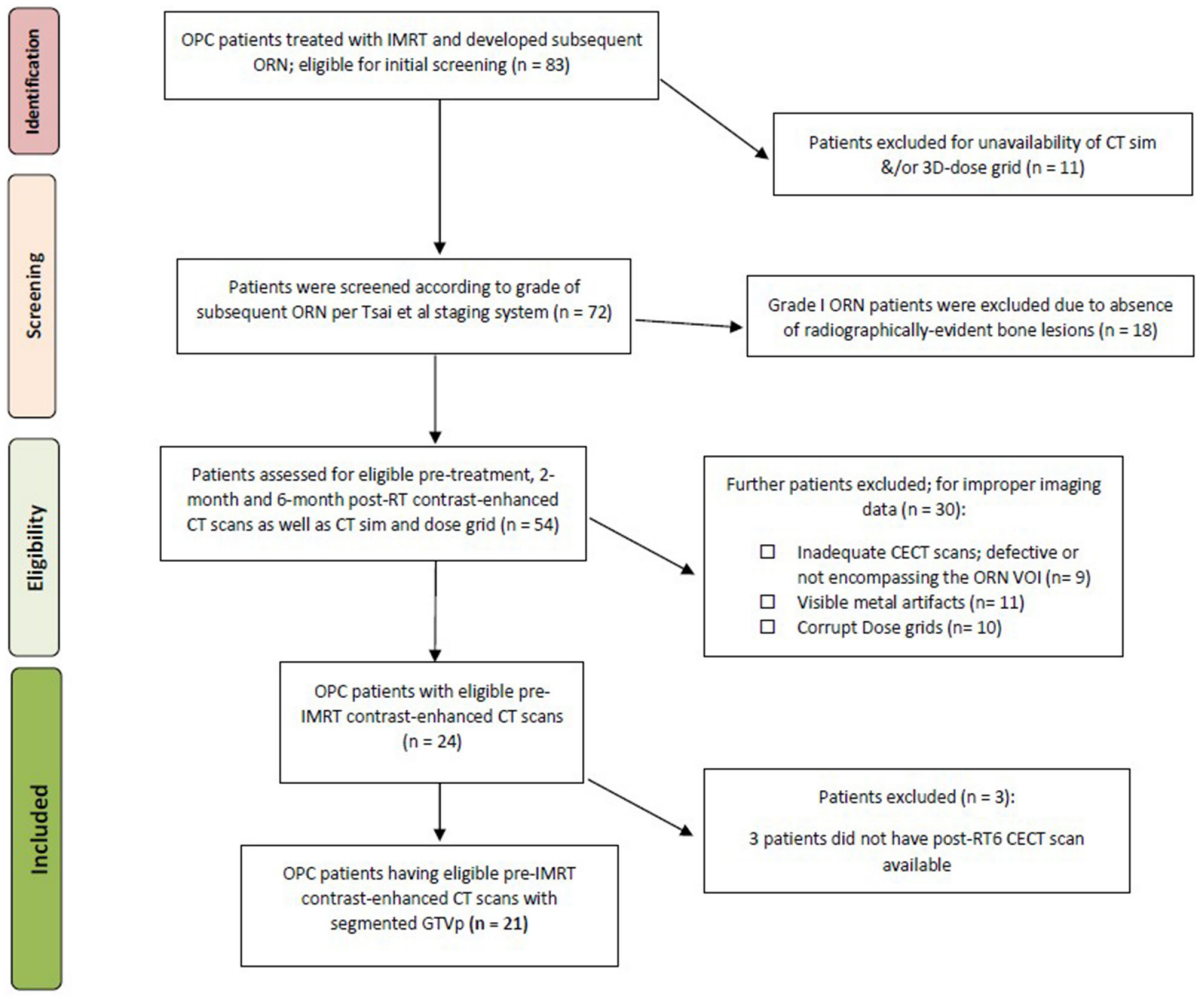
Patient selection. Flowchart of selection process of patients for this study.

Following approval from an institutional review board (IRB) at our institution, data for biopsy-proven OPC patients treated between 2002 and 2013 who underwent radiation therapy as a single or multimodality definitive therapy were considered for the current investigation (*n* = 83). This investigation and relevant methodology were performed in compliance with the Health Insurance Portability and Accountability Act (HIPAA) as a retrospective study where the need for informed consent was waived (Freymann et al., 2012). Electronic medical records were scanned for documented diagnosis of mandibular ORN following IMRT in the absence of any prior head and neck re-irradiation along the same lines as a previous ORN study by our team (Mohamed et al., 2017). The aspects of our institutional IMRT approach for oropharyngeal cancer patients were previously reported in detail (Garden et al., 2013). All patients received pre-radiotherapy Dental Oncology service clearance, and, if indicated, prophylactic dental extraction and fluoride trays were prescribed as per standard Head and Neck Service operating procedure (Tsai et al., 2013). Inclusion and exclusion criteria for patients' selection are illustrated in Figure 1.

### ORN Staging

The severity of ORN was graded I through IV as follows: grade I, i.e., minimal bone exposure requiring conservative management; grade II: minor debridement required; grade III: hyperbaric oxygen therapy (HBOT) received; grade IV: major surgery mandated. This staging system is very comprehensively given its emphasis on response to treatment as a standard to categorize ORN (Tsai et al., 2013). Patients who subsequently suffered from radiographically &/or pathologically proven grade II or worse ORN were included in this study.

### CT Acquisition Protocol and Eligibility Criteria

According to our institutional protocol, CECT images were obtained as a prerequisite for pre-treatment diagnostic work-up. Subsequent post-IMRT CECT scans for response evaluation and further surveillance were routinely performed at 2 and 6-month time points and then at regular preset intervals thereafter. Our study revolved about extracting quantitative imaging biomarkers from CECT at pre-IMRT (i.e., baseline), 2-month (post-RT2), and 6-month (post-RT6) post-IMRT, as well as the time instance corresponding to the development of ORN. To that end, CECT scans with available non-reconstructed axial cuts at the aforementioned 4 time points were retrieved. CT slices with evident ORN lesions that were obscured or otherwise affected by visible metal artifacts were not contoured and were not included in the analysis.

All CT scans were attained with a multi-detector row CT scanner. Scan parameters were as follows: slice thickness reconstruction (STR) ranges between 1 and 3 mm, with a median STR of 1 mm, X-ray tube current of 99–584 mA (median: 220 mA) at 120–140 kVp. All images acquired at our institution were composed of 512 × 512 pixels and were acquired following a 90 s delay after intravenous contrast administration. One-hundred and twenty milliliters of contrast were injected at a rate of 3 ml/s. To standardize the image voxel sizes for use in texture feature calculations, all the CT scans were resampled, via a trilinear interpolation voxel resampling filter (Shafiq-ul-Hassan et al., 2017).

### Image Segmentation and Registration

We specifically selected CECT scans demonstrating the earliest radiographically evident ORN characteristic lesion(s) as reported by radiologists and further confirmed by physical examination by physicians in Head & Neck Surgery as well as in Dental Oncology [ORN CECT].

The original delivered DICOM-RT clinical treatment plans were restored from Pinnacle treatment planning system (Pinnacle, Phillips Medical Systems, Andover, MA) into commercially available image registration software (VelocityAI™ 3.0.1). Diagnostic CECT scans at baseline, post-RT2, post-RT6, and ORN were also imported. Radiographically evident bony lesions were delineated manually by a radiation oncologist (HE) to constitute the ORN volumes of interest (VOIs). Physical exam and other available imaging modalities such as dental-dedicated panoramic X-rays were utilized to guide the segmentation of VOIs.

**Figure 2:**
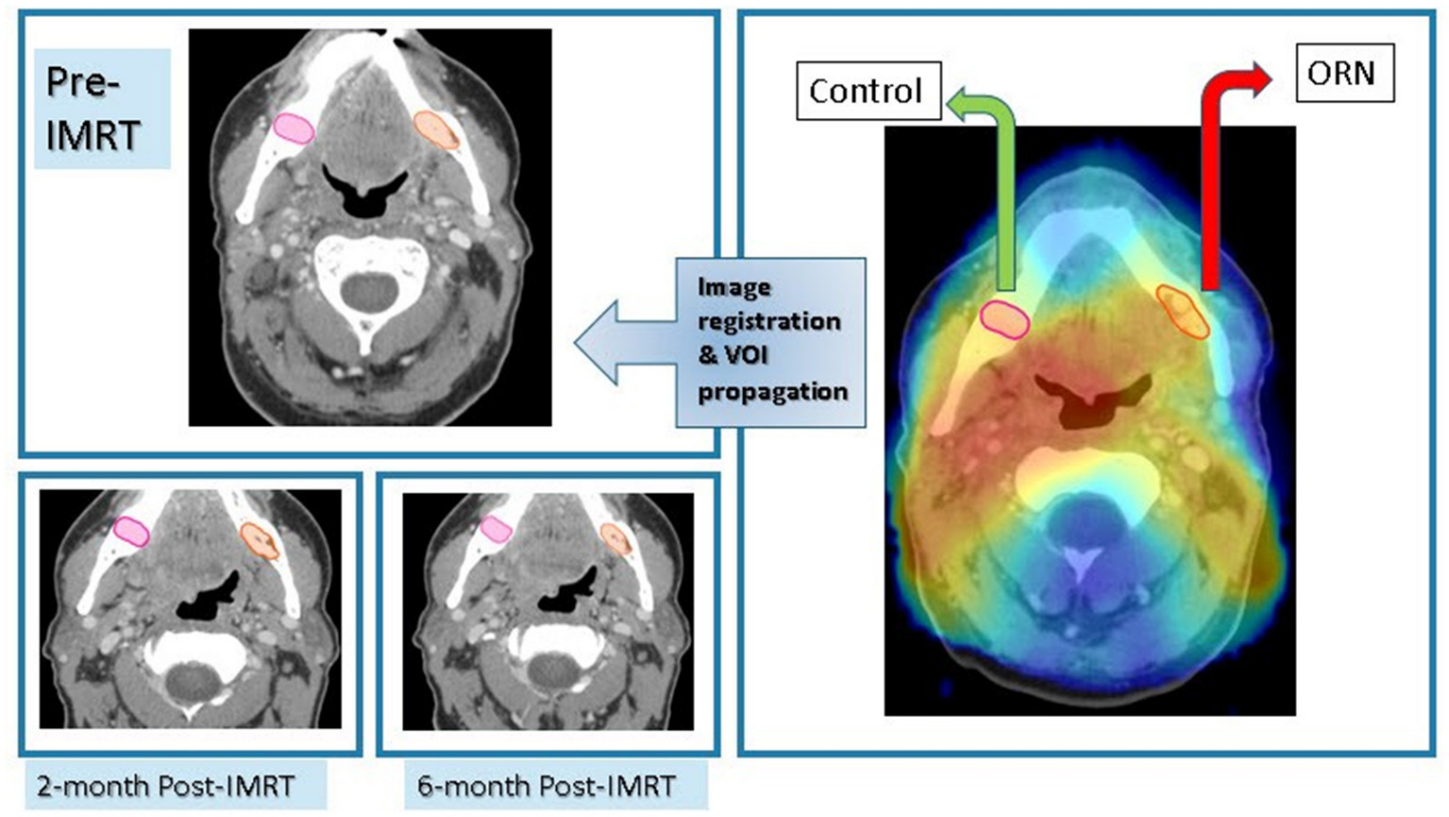
Imaging workflow. Registration of CECT scan at time of diagnosis of ORN to radiation dose grid as well as previous CECT scans at: baseline, 2-month, and 6-month post-RT for each patient with subsequent propagation of ORN & “Control” VOIs.

Planning CT was co-registered with ORN CECT using deformable image registration algorithm of VelocityAI™ 3.0.1. The 3D reconstructed dose grid of RT plan was then overlaid to the ORN CECT. A neighboring radiographically intact mandibular subvolume within the same isodose distribution volume was manually segmented and designated as “Control VOI” at the ORN CECT. Subsequently, baseline, post-RT2, post-RT6 CECT scans were co-registered with ORN CECT using rigid registration algorithms of VelocityAI™ 3.0.1. Both “ORN” & “Control” VOIs were propagated from ORN CECT to other CECT scans at all three prior time points (Figure 2).

### Radiomics Features Extraction

Computed tomography scans with corresponding contoured VOIs were then extracted in the Digital Imaging and Communications in Medicine format (DICOM), as DICOM-RT and RT-STRUCT files, respectively. These files were then imported into an in-house image biomarker explorer (IBEX) software, built on MATLAB for subsequent radiomics feature extraction (Zhang et al., 2015) along the same lines as previous studies (Elhalawani et al., 2018; Yang et al., 2018).

Radiomic features were derived from two VOIs that correspond to ORN and Control in the 3 prior time points: pre-IMRT, post-RT2, and post-RT6 CECT scans. The number of radiomic features extracted for each VOI summed up to 1,645 individual features. They included a myriad of first- and second-order radiomic features (Supplementary Table 1). Second-order radiomic features were calculated in both full 3-dimensional images (3D) as well as 2.5D, i.e., features calculated for each 2-dimensional slice and results were then combined. Other than shape features, a trilinear interpolation voxel resampling filter to 3 mm slice thickness and 1 mm^2^ pixel spacing was applied prior to feature extraction to standardize voxel size. First-order feature categories include shape, intensity direct, and intensity histogram. Whereas second-order feature categories encompass: Gray level co-occurrence matrix (GLCM), gray level run length matrix (GLRL) as well as neighborhood intensity difference. For GLCM and GLRL features, calculations from multiple spatial directions were combined to produce one value (Materka and Strzelecki, 1998). For NID, 3 different permutations of neighborhood, i.e., 3, 5, or 7 were employed as in previous projects (Elhalawani et al., 2018,a,b).

### Radiomics Features Pre-selection and Reduction

Initially, we worked with radiomic features computed from VOIs corresponding to ORN and Control for 24 patients. The number of radiomic features extracted for each patient is 1,628. Three patients did not have radiomic features computed for the post-RT6 time point and hence completely excluded from subsequent analysis. For these 21 patients, we only kept the radiomic features whose values are available for (i) all 3 time points, and (ii) both in “ORN” and “Control” VOIs. One patient has 2 distinct ORN lesions; accounting for a total number of 43 individual VOIs (22 “ORN” and 21 “Control” VOIs). Thus, we are then left with 1,628 radiomic features from 43 VOIs, i.e., 22 “ORN” and 21 “Control.”

Feature reduction by correlation was critical to ensure that the performance of any machine learning algorithm is not degraded because of a high degree of correlation in the features, or multicollinearity (Garg and Tai, 2013). We first compute the Spearman correlation (Landberg et al., 1999; Zar, 2005) for the 1,628 radiomic features at the pre-IMRT time point. We filter out the features whose average correlation level with all the remaining features is greater than a user-defined threshold (Kuhn, 2008). For our data, we used a threshold of 0.5. The threshold was chosen to reasonably balance the dual requirements of multicollinearity reduction and capturing data variation. Following correlation filtering, we reduced the number of features we analyze to 16 features (Supplementary Table 2).

First—as a proof of concept—, we sought to establish that radiomics can quantitatively discriminate between ORN and non-ORN mandibular subvolumes. Mann-Whitney test (Mann and Whitney, 1947) was used to identify specific radiomic features that show statistically significant differences between ORN and non-ORN high-risk VOIs.

### Functional Principal Component Analysis

We hypothesize that we can predict the risk of ORN by looking at the temporal evolution of radiomic features. A standard way of identifying temporal signatures in time series data is by using functional principal component analysis (FPCA) (Shang, 2014; Aue et al., 2015). FPCA takes multiple time series curves, as an input, and tries to find the underlying shape signatures that optimally can be used to represent all the curves. These shape signatures are called the functional Principal Components (PC). Each time series can now be represented by a weighted combination of each of the PCs. This technique has been used to predict outcomes from sequential data in a wide variety of fields such as remote sensing (Cardot et al., 2003), stock markets (Foutz and Jank, 2010), electroencephalogram (EEG) analysis (Shou et al., 2015), and cancer pathology (Barua et al., 2018). Since our data is multivariate, in that we have a time series for multiple features for the same patient, we can compute the functional PCs for each feature. One way of representation would be to assume each feature is independent, concatenate the PC weights for each feature, and use this concatenated representation as input to a machine learning model. However, since each pair of features is correlated to various degrees, we use a technique called multivariate FPCA (MFPCA), which explicitly accounts for the relationship between the features (Dauxois et al., 1982; Berrendero et al., 2011; Chiou et al., 2014; Happ and Greven, 2018). We utilized the R package MFPCA for our temporal kinetics analysis (Happ and Greven, 2018).

**Figure 3:**
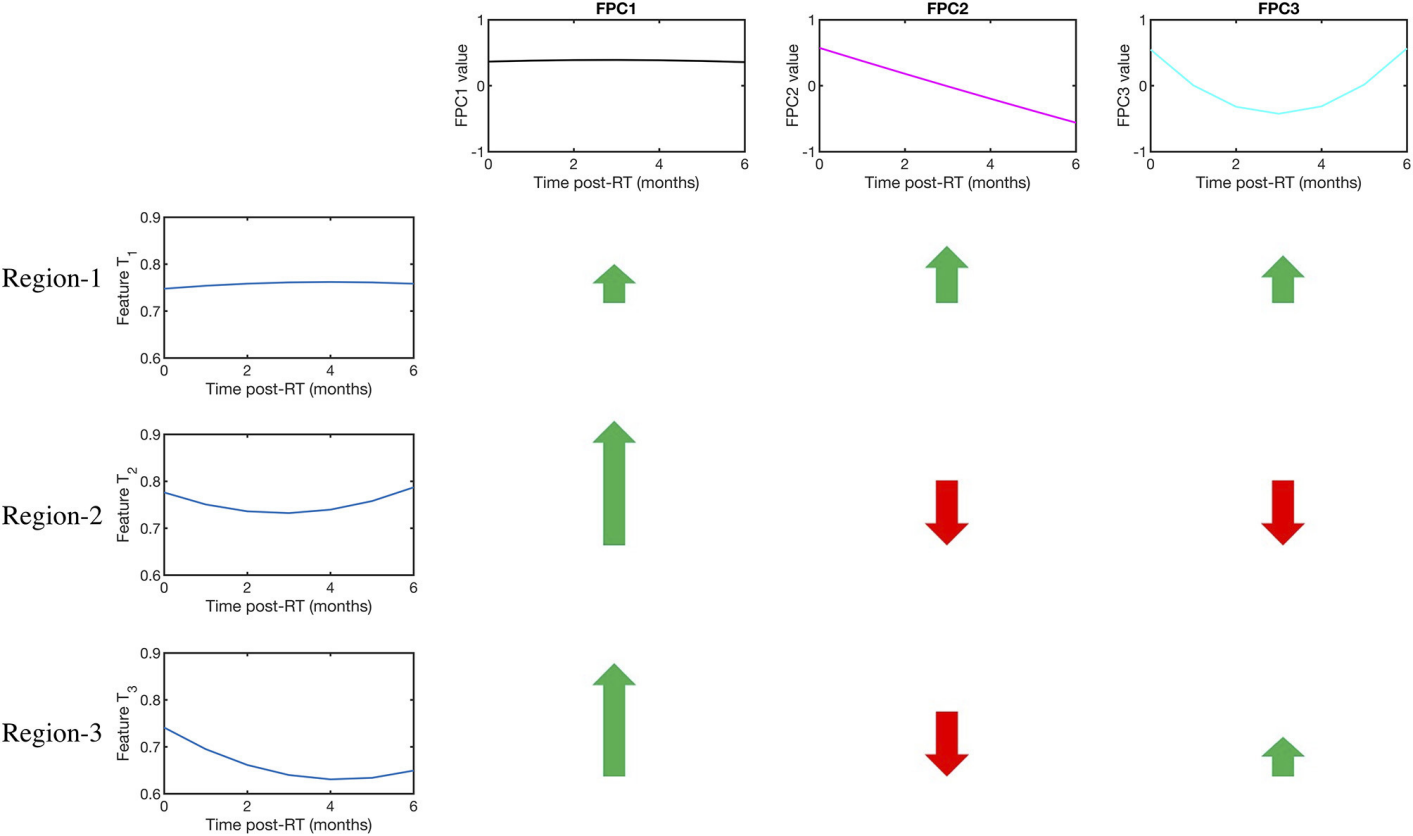
Visual explanation of the FPCA algorithm and its advantages. The first row displays the 3 functional principal components (FPCs). On the left column, the temporal evolution of a Gray Level Co-occurrence Matrix (GLCM)-3D feature is shown for three mandibular regions namely Regions 1,2, and 3. Regions 1 and 2 did not develop ORN, while 3 did. We note that Regions 1,2, and 3 all have similar baseline values, so cannot be distinguished by a model built solely on pre-radiotherapy features. Further, Regions 2 and 3 also have similar change in their values, which a delta radiomics model would see as equivalent scenarios. On the other hand, the difference in the temporal kinetics is efficiently encoded in the 3 FPCs. The color and length of the arrows indicate the sign (+ve or –ve) and magnitude (large or low) of relative contribution made by each FPC in explaining the time series. So, for example, Region 2 and Region 3, which appear alike to a pre-radiotherapy model and a delta radiomics model, can be readily distinguished because of the difference in relative contribution made by the 3rd FPC.

The importance of FPCA is visually explained in Figure 3. We display 3 temporal trajectories from our data on the leftmost column. We observe that all 3 sequences *T*_1_, *T*_2_, and *T*_3_, have similar starting points. Further time series *T*_2_ and *T*_3_ have similar end points too. This mimics a significant scenario which we try to address, whereby neither the pre-radiotherapy features, nor the delta features can distinguish between the patients. However, FPCA can distinguish all 3, by accounting for both, the values taken by the time series, and the shape of the trajectory. The top 3 FPCs representing the dataset are shown visually in the top row. The relative contribution of each FPC to each of the time series is shown with arrows, the length of the arrows representing the magnitude, and green and red color indicating the sign (positive and negative, respectively) of the contributions. We can see that the magnitude and sign of the individual contributions from the PCs are quite different, and thus can help distinguish the three-time series.

### Training the Random Forest

We used repeated random sampling to produce random forests where validation (Breiman, 2001) ensued where validation of each forest was performed using the left out observations, and the overall accuracy was calculated by averaging the class predictions of each of the forests. The random forest has been shown to be robust to over-fitting and among the most effective of the commonly used classifiers (Breiman, 2001). Each forest used 500 trees, and each split was determined using $\sqrt{p}$ features where *p* is the number of features. The random forest calculations were performed using the random Forest package for R software (Liaw and Wiener, 2002). To further examine the performance of the model, the ROC curves were plotted and the area under the curve (AUC) was calculated using pROC package for R (Robin et al., 2011).

## Results

### Patient Information

Twenty-one patients with oropharyngeal cancer (OPC) were identified to have developed ORN after their definitive radiotherapy ± chemotherapy course, either in induction and/or concurrent settings as in Figure 1. Eight patients developed grade 2 ORN, whereas 2 patients and 11 patients developed grade 3 and 4 ORN, respectively. The median time to ORN diagnosis was 20.3 months. Table 1 represents patient demographics, tumor, radiation dose, and ORN disease characteristics.

**Table 1:** Patients, disease, and treatment characteristics.

**Characteristics**	***N* (%)**
**SEX**	
Male	20 (95.2%)
Female	1 (4.8%)
Age at diagnosis, years: median (range)	61 (57–68)
**ETHNICITY**	
White or Caucasian	17 (81%)
Hispanic or Latino	2 (9.5%)
African American	2 (9.5%)
**SMOKING STATUS**	
Current	10 (47.6%)
Former	5 (23.8%)
Never	6 (28.6%)
Smoking pack-years (median; IQR)	10 (0–40.5)
**TUMOR LATERALITY**	
Right	9 (42.9%)
Left	11 (52.4%)
Midline	1 (4.7%)
**OROPHARYNX SUBSITES**	
Base of tongue	12 (57.1%)
Tonsil	7 (33.3%)
NOS*	2 (9.6%)
**T CATEGORY**	
T1	2 (9.5)
T2	10 (47.6%)
T3	5 (23.8%)
T4	4 (19.1%)
**N CATEGORY**	
N0	2 (9.5%)
N1	0
N2	19 (90.5%)
N3	0 (0)
**THERAPEUTIC COMBINATION**	
Induction chemotherapy (IC) followed by concurrent chemoradiation	10 (47.6%)
IC followed by radiation alone	1 (4.8%)
CC	10 (47.6%)
**VITAL STATUS**	
Alive	14 (66.7%)
Dead	7 (33.3%)
Radiation dose (median; IQR) [Gy]	70 (66–70)
Radiation fractions (median; IQR)	33 (30–33)
Onset of post-RT ORN (median; IQR)	20.3 (7.5–95)
**ORN LATERALITY (IN RELATION TO PRIMARY TUMOR)**	
Ipsilateral	17 (81%)
Contralateral	2 (9.5)
Bilateral	2 (9.5%)
**RADIATION DOSE AT THE ORN VOLUME (MEDIAN; IQR) [GY]**	
Mean dose	67.9 (59.5–71.2)
Minimum dose	51 (44–59.4)
Maximum dose	68.9 (67.6–73.1)

*IQR, inter-quartile range; Gy, Gray; NOS, Not otherwise specified; ORN, osteoradionecrosis*.

#### Radiomics Can Distinguish Between ORN and Non-ORN

An initial set of 1,628 radiomic features were computed for each ORN and Control volume of interest (VOI) obtained from the 21 eligible patients across 3 time points of interest representing baseline (pre-IMRT), 2-month (post-RT2) post-IMRT, and 6-month (post-RT6) post-IMRT. Sixteen radiomics features were ultimately nominated as non-interrelated and consistently available for all three time points. As an initial exploratory step, we computed which of these 16 radiomic features were significantly different between the ORN and Control volumes of interest (VOIs) using a Mann-Whitney test. Furthermore, we also computed if each of these features is larger, or smaller, on average for the ORN VOI compared to the Control VOI. This demonstrates that certain radiomic features differ significantly between ORN and non-ORN regions, motivating us to investigate if their evolution can foretell ORN incidence. The significantly different features and their associated *p*-values are reported in Table 2.

**Table 2:** Significantly differing radiomics features between ORN and Control VOIs.

**Feature**	***p*-value**	**Mean difference of feature value between ORN and Control feature values**
Gray Level Co-occurrence Matrix 25-333-1 InformationMeasureCorr1	0.028	Negative
Gray Level Co-occurrence Matrix 312-4 Cluster Shade	0.034	Positive
Gray Level Co-occurrence Matrix 310-1 Dissimilarity	0.009	Positive
Gray Level Co-occurrence Matrix 38-1InverseDiffMomentNorm	0.0002	Negative
Intensity- Mean	2.43E-7	Negative
Intensity- Local entropy median	4.65E-6	Negative

The radiomics features which values are significantly different between the “ORN” and “Control” VOIs at the ORN time point identified using a Mann-Whitney test. The corresponding *p*-value is reported in the second column. We also report the direction of the difference of means between the ORN and Control VOI feature values in the third column.

#### Model Construction

**Figure 4:**
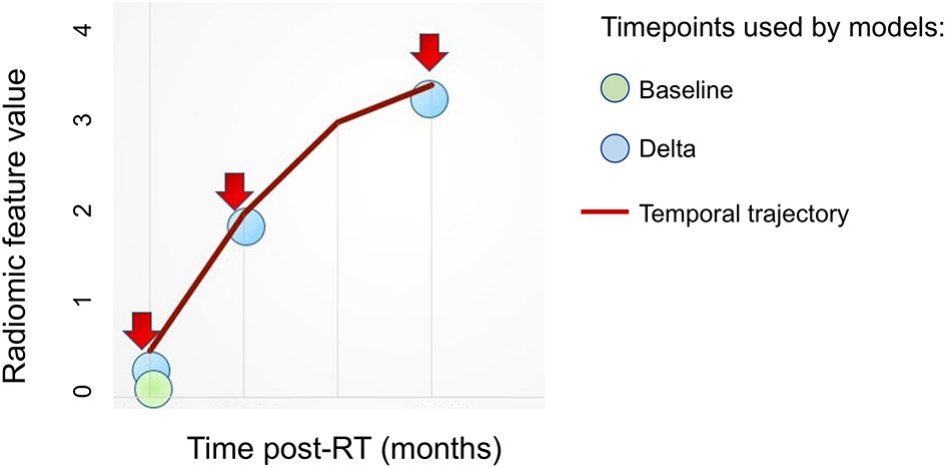
Overview of radiomics features based approaches. Various approaches to integrate radiomics features obtained at multiple (≥1) time points toward building predictive models.

We trained random forest models using 500 trees for each of multiple approaches as outlined below: (Figure 4)

- **Baseline:** Radiomic features computed on the pre-IMRT CECT scans.
- **Delta (2-month follow-up):** Relative change in the radiomic features from pre-IMRT to post-RT2
- **Delta (6-month follow-up):** Relative change in the radiomic features from pre-IMRT to post-RT6.
- **Temporal Trajectory:** The model built using the proposed multivariate functional principal component analysis (MFPCA) approach that models the temporal kinetics of the features. Since the time points are not uniformly spaced, we used cubic spline sequence completion to fill in radiomic features at intermediate monthly time points.
- **Baseline** **+** **Temporal Trajectory:** We combined the predictions from the baseline model and the temporal trajectory model to give a more robust ORN-risk predictor.

A complete step-by-step guide for the model construction pipeline is presented in Appendix A1.

**Figure 5:**
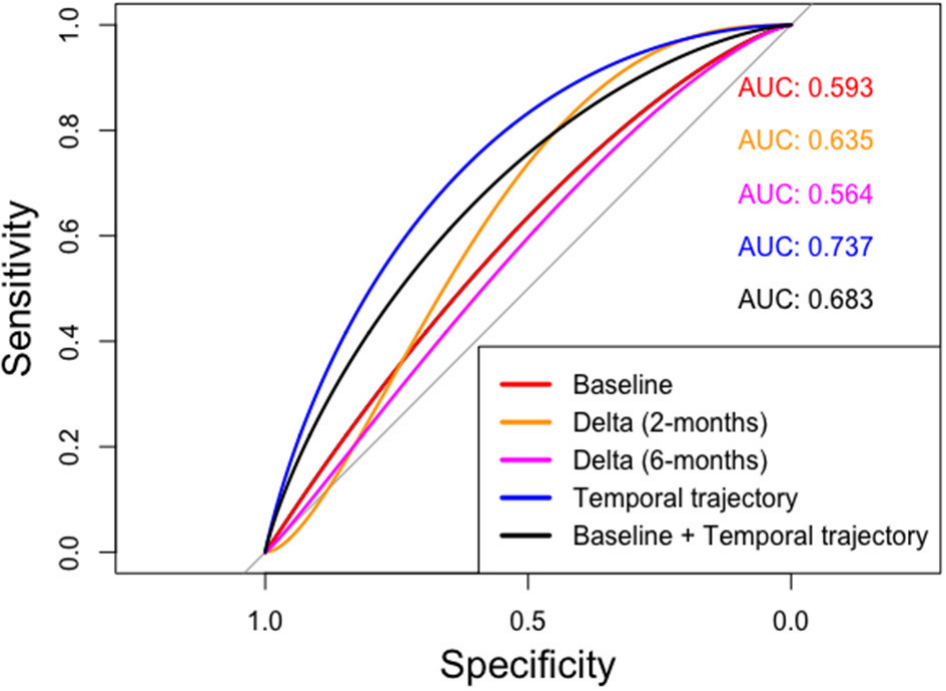
ROC curves computed for various radiomics feature based approaches. The temporal trajectory model using MFPCA (blue) performs better than the other four models: (i) baseline model (red), (ii) delta model after 2-month follow-up (orange), (iii) delta model after 6-month follow-up (purple), and (iv) an ensemble of baseline and temporal trajectory models (black).

The corresponding areas under the curves (AUCs) and 95% confidence intervals (C.I.) for the prediction of occurrence of ORN “Yes vs. No,” in both “ORN” and “Control” VOIs according to the 5 models are depicted in Table 3 and illustrated in Figure 5. We noticed that the baseline features model gives an AUC of 0.59 (95% C.I: 0.41–0.76), while the temporal trajectory gives an AUC of 0.74 (95% C.I: 0.61–0.9). We further built an ensemble model that combines the predictions of the baseline model and the temporal trajectory model, to see if these two models have complementary information that improves performance. We achieved an AUC of 0.68 (95% C.I: 0.53–0.86), likely due to the poor performance of the baseline model which consequently was detrimental to the performance of the combined model. This suggests a more careful approach is needed when choosing pre-IMRT features. Surprisingly, models constructed using percent changes “or delta changes” of the radiomic feature values, performed poorly in predicting ORN incidence with AUCs of 0.64 (95% C.I: 0.46–0.81) and 0.56 (95% C.I: 0.39–0.74) for 2 and 6-month delta changes, respectively. We further observe that the temporal trajectory and combined models have a consistent performance in both low-specificity and high-specificity regimes, in contrast to the delta models which performance is dependent on the regime of choice. This demonstrates that greater reliability is achieved by incorporating the temporal kinetics of the radiomic features. We do note that as a result of the small sample size, the confidence intervals of the models are wide and overlapping. As such, larger validation studies are needed to gauge the true performance of the models.

**Table 3:** A comparison of the Areas under the curves (AUCs) and the 95% confidence intervals for the various approaches.

**Method**	**AUC (95% CI)**
Baseline	0.59 (0.41–0.76)
Delta (2-month follow-up)	0.64 (0.46–0.81)
Delta (6-month follow-up)	0.56 (0.39–0.74)
Temporal trajectory	0.74 (0.61–0.90)
Baseline + Temporal trajectory	0.68 (0.53–0.86)

To enable the use of our temporal trajectory model for the stated aim of ORN prediction, we compute the optimal point on the ROC curve as the point that maximizes the Youden's index (sensitivity+specificity-1) (Youden, 1950). As shown in Supplementary Figure 1, the optimal point corresponds to a sensitivity of 0.73 and specificity of 0.62. The optimal threshold for the temporal trajectory model, which represents the cutoff probability value above which a given mandibular region is predicted to be “Control” is found to be 0.54. Thus, if the temporal trajectory model predicts the likelihood of a given region as lower than 0.54, the region is classified as “ORN” and if the probability is higher, the region is classified as “Control.” We next generate the confusion matrix for the 43 regions classified using the optimal threshold value; 72.7% of ORN regions and 61.9% of Control regions were correctly classified as shown in Supplementary Figure 2.

## Discussion

The incidence of head and neck cancer is on the rise, despite reductions in smoking, owing to the recent prevalence of the human papillomavirus (HPV)-associated OPC epidemic (Ang et al., 2010). Forward, it's projected that hundreds of thousands of locally advanced OPC patients worldwide will receive radiation to the head and neck as a definitive treatment modality (Chaturvedi et al., 2011). This rise in RT recipients implies that mandibular bone, which comprises the borders of the oropharynx, will be necessarily irradiated to ensure adequate tumor coverage with subsequently growing incidence of crippling sequelae such as ORN (Gomez et al., 2011).

Osteoradionecrosis ranges from superficial, slowly progressive bone erosion/devitalization to pathological fracture in a previously irradiated field and may cause significant hardship in the afflicted individual (Mendenhall, 2004; Hamilton et al., 2012). This is particularly apparent when considering devastating lifelong issues with oral hygiene, nutritional inadequacies, and difficulty with speech and resultant preclusion of social interaction (Bonner et al., 2006). Early diagnosis and intervention, whether conservative or surgical, are key for improving outcomes (Ben-David et al., 2007). This essentially applies for grade II ORN, where no consensus has been reached regarding definitive treatment procedures (Oh et al., 2009; Jacobson et al., 2010).

### Using CT Radiomics to Identify Mandibular Subvolumes At-Risk of ORN

To date, no imaging modality/clinical nomogram have been shown to precisely foresee the potential risk of developing osteoradionecrosis following IMRT (Allison et al., 2014). Being fully integrated throughout various phases of HNC management, sequential CECT scans via radiomics analytics can provide a plethora of data that can serve as quantifiable surrogates of tissue vitality and vascularity, among others (Wong et al., 2016). To our knowledge, this study is the first to characterize the kinetics of radiomics features of various mandibular subvolumes, before and after exposure to IMRT, to identify subvolumes at high risk ahead of developing ORN. Radiomics features were analyzed longitudinally for quantifying temporal changes in mandibular bone structure in a cohort of OPC patients.

### Applying FPCA to Capture Longitudinal Changes in Mandibular Radiomic Features

This has been subsequently integrated into a framework for early prediction of ORN solely based on sequential diagnostic CECT scans. We implemented a Functional Principal Component Analysis (FPCA)-based approach that efficiently models the temporal evolution of radiomic features. The model built using a multivariate FPCA (MFPCA) representation of the entire temporal dataset, predicts the likelihood of ORN development with an AUC = 0.74 (95% C.I 0.61–0.9). We further built an ensemble model that combines the predictions of a baseline model built using pre-IMRT features, and the MFPCA-based model, to leverage information from both baseline feature values and temporal evolution of feature values, which achieved an AUC of 0.68 (0.53–0.86). This emulates the pathophysiology theories that combine pre-irradiation bone condition and RT-induced alterations on tissue, cellular and cytokine levels (Fan et al., 2014). The latter involves: (1) endarteritis and vascular thrombosis with subsequent bone hypoxia and hypocellularity as well as atrophic fibrosis as a consequence of RT-induced activation and dysregulation of fibroblastic activity (Marx, 1983; Jacobson et al., 2010). The fact that the ensemble model does not perform better than the MFPCA-only model suggests the need to choose the pre-IMRT features in a way that is more clinically meaningful than a purely data-driven correlation thresholding approach.

Bone texture analysis has been investigated for years as a potential biomarker of a myriad of structural bone changes related to osteoporosis (Ollivier et al., 2013; Roberts et al., 2013). Interestingly, first-order bone texture features derived from simulation CT scans were correlated to the risk of radiation-induced insufficiency fractures in patients undergoing pelvic radiation (Nardone et al., 2017). Along the same lines, for vascularization status, a previous study by Yin et al. investigated the correlation between angiogenesis (or: new blood vessel formation) in primary renal cell carcinoma and radiomic imaging features from positron-emission tomography (PET) and/or MRI (Yin et al., 2017).

Our study identifies the bone radiomics features which temporal evolution is critical in determining ORN risk. These represent quantifiable imaging biomarkers that capture various intensity and spatial texture dimensions of the aforementioned RT-related bone environment changes in the irradiated field. Most of the discriminating features belong to: “Neighborhood intensity difference” (NID) and “Gray level co-occurrence matrix” (GLCM) categories. The GLCM is a matrix that expresses how combinations of discretized gray levels of neighboring pixels, or voxels in a 3D volume, are distributed along one of the image directions. Generally, the neighborhood for GLCM is a 26-connected neighborhood in 3D and an 8-connected neighborhood in 2D (Liang et al., 2016). The “NID 2.5D Texture strength” quantifies how uniform a texture is, i.e., complex textures are non-uniform and rapid changes in gray levels are common (Amadasun and King, 1989). GLCM3 Cluster shade is a measure of the skewness or asymmetry of the matrix and is believed to be a more objective uniformity metric (Unser, 1986). On the other hand, GLCM3 Contrast gauges gray level variations in the volume of interest, i.e., the difference between the highest and the lowest values of a continuous set of pixels (Haralick et al., 1973). GLCM3 Correlation is a measure of texture smoothness, where higher values denote regions with similar gray-levels (Yang et al., 2012). Nonetheless, it is unclear how these radiomic features are linked to well-known physiological underpinnings of ORN evolution. A future validation study including biological imaging is warranted to investigate the link between these radiomic features and physiological properties.

We have seen that there is significant information regarding ORN progression in the first 6 months after radiotherapy that can be robustly correlated to risk of ORN. Functional principal component analysis is an efficient statistical algorithm to capture the temporal evolution of the mandible landscape. Competing techniques such as pre-radiotherapy only models and delta radiomics models do not encapsulate how different features evolve with time. The FPCA efficiently encodes the temporal kinetics of the features into its functional principal components (FPCs). The radiomics data can now be compactly represented by only a small set of numbers but can still capture its time-varying properties.

Furthermore, we implement a multivariate FPCA (MFPCA) that accounts for the correlations that exist between various radiomics features. MFPCA distills a large set of features to a few specific ones that encompass most of the data variation. This makes our prediction model more likely to generalize to new, unseen data (Happ and Greven, 2018). We observe from the receiver operating characteristic curves that the temporal trajectory model performs consistently better than the other models in both the high- and low-specificity or false positive regions. This demonstrates the reliability of using temporal kinetics, for example, compared to a delta model, which we observed to have vastly different performance depending on the specificity value. The combined prediction model does not improve over the temporal trajectory only model, possibly because of the extremely poor performance of the baseline model. However, the combined model also performs consistently in both the low- false positive or high false positive regimes. We envisage that with a more careful choice of features, the baseline model can be improved, which will significantly improve the performance of the combined model. We note however that while the average performance of the MFPCA model is at least 10 percentage points better than either the baseline or the two delta models, the respective confidence intervals are overlapping across models. As such, larger validation studies are needed to find out the true predictive ability of each of the models investigated.

The preliminary feature filtering step was performed by setting an upper limit of 0.5 on average correlation value for a given radiomic feature. Meaning, if a given radiomic feature correlated with all other features more than 0.5 on average, it was dropped from our feature set. The choice of value was made to whittle the number of features down from a mammoth 1,628 to a more manageable 16 given the small sample size of our cohort. The reduction of features is necessary to compute robust functional principal components as well as reduce the possibility of overfitting by the random forest models. We also found that reducing the number of features further led to a drop in the model performance, which suggests loss of information crucial to prediction performance.

Our study accounted for the fact that artifacts from metal dental fillings are known to encumber target delineation and subsequent radiomics analysis (Leijenaar et al., 2015; Block et al., 2018). For this purpose, the presence of visible dental artifacts effect anywhere in the slices that encompassed “ORN” or “Control” VOIs at any time point precluded the integration of this scan and hence the patient's data as an input to the model.

### Study Limitations

The fact that we excluded these patients with metal dental fillings, combined with the low event rate of ORN in the IMRT era, as well as the fact that we excluded patients with grade I ORN with no radiographically-evident bone lesions to delineate, contributed to the low sample size; hence limiting the generalizability of the resulting model. The small sample size limited us to apply automatically generated radiomics features instead of engineering features that are explicit surrogates for early vascular injuries of the mandible. Sub-group analysis based on variables such as T-stage, radiation dose, and chemotherapy usage were infeasible because smaller sample sizes within each group reduces the robustness of the functional principal components computed and hence the statistical value of any subsequent sub-group analysis. Another limitation of this study is the conceivable uncertainties introduced from varied acquisition parameters or incongruence among various scanners, or even between different models from the same vendor (Mackin et al., 2015). Most patients had their scan performed at our center along the same acquisition parameters. Moreover, we have applied a pre-processing trilinear interpolation aiming at standardizing voxel size to reduce or eliminate relevant variability in radiomics features (Mackin et al., 2017). The results also suggested that the performance changed rapidly when we changed the number of features, which suggests the need for a more careful feature-filtering algorithm. Designating a “Control” VOI that share the same image, time point, and deposited radiation dose with the “ORN” VOI is an approach we have used and would recommend for future multi-institutional radiomics studies. However, it should be noted that our model was trained on a homogenous, carefully selected set of patients with OPC where mandibles received similarly high doses of radiation; hence limiting model generalization to varying clinical scenarios.

### Future Directions

Not far from longitudinal imaging studies, our team previously showed that Dynamic Contrast-Enhanced (DCE-MRI) can provide biomarkers that are physiological correlates of acute mandibular vascular injury and recovery temporal kinetics (Joint and Neck Radiotherapy, 2016). This has further motivated a National Institute of Dental and Craniofacial Research (NIDCR)-funded prospective trial that explores the correlation between DCE-MRI derived spatiotemporal parameter maps following external beam radiation therapy (EBRT) and subsequent development of ORN (ClinicalTrials.gov S., 2020). Upon accrual completion, CT scans from this study will be used for re-training and externally validating our model. This could potentially optimize model generalization since patients will display more diverse and representative head and neck cancer sites, radiation doses, and other clinical variables. Our results may prompt the investigation of DCE-MRI-derived radiomics analytics and subsequent integration into the overall predictive model; thus, providing more physiologically and biologically cognizant data inputs for the machine learning techniques tested.

Furthermore, the availability of larger cohorts will provide potential avenues for model validation and generalization over the whole mandible in patients with ORN vs. healthy controls. Specifically, a larger cohort would make it possible to examine the performance of our FPCA-based model across T-stage, radiation dose, and chemotherapy usage, providing additional insights into the impact of these variables on ORN development. In future validation studies, we plan to enroll more patients with more evenly distributed variable levels. A proposed application would be engineering radiomics features that are explicit surrogates for osteoclastic dysregulation and subsequent fibro-atrophic bone changes, and maybe monitoring the response to common therapeutic maneuvers, such as pentoxifylline.

## Conclusion

Radiomics analysis allows for quantification of changes in RT-related bone structure from diagnostic imaging modalities with subsequent integration of serially derived radiomics features into an ORN probability computational tool. Computationally, FPCA efficiently encodes the temporal kinetics of a given radiomic feature. The MFPCA then compactly combines the temporal information from FPCA from multiple radiomic features.

In summary, we hope this study calls professionals' attention to non-traditional inputs (radiomics), dimensions (temporal kinetics), and innovative statistical approaches (MFPCA) to improve interpretation and integration of imaging biomarkers into RT toxicities prediction and mitigation. In this work, we have thus provided an end-to-end framework for predicting the risk of RT-related ORN based entirely on radiomic features.

## Data Availability Statement

Clinical dataset is not available as it includes personal health identifiers (PHI). It is possible for de-identified data to be made available upon reasonable request.

## Ethics Statement

The studies involving human participants were reviewed and approved by MD Anderson IRB RCR-03-800. Written informed consent for participation was not required for this study in accordance with the national legislation and the institutional requirements.

## Author Contributions

All authors performed substantial contributions to the conception or design of the work, or the acquisition, analysis, or interpretation of data for the work, drafting the work or revising it critically for important intellectual content, final approval of the version to be published, agreement to be accountable for all aspects of the work in ensuring that questions related to the accuracy or integrity of any part of the work are appropriately investigated and resolved. SB and HE: manuscript writing, conceived study idea, and designed workflow. SB designed the temporal kinetics software framework to filter radiomics features and compute FPCA, implemented the random forest models to compare pre-IMRT, delta, and FPCA models, and conducted statistical analyses. HE: direct oversight of image segmentation/image post-processing, supervision of DICOM-RT analytic workflows and clinical data collection workflows. SV, KA, PY, SN, and BE: electronic medical record screening, data extraction, image segmentation/registration, and clinical data collection. RG: Informatics software support. MC: Database construction, clinical/oncologic oropharynx database curation and oversight, conceptual feedback, and support. KH, and GG: data provision, patient case extraction, supervisory support, editorial oversight, programmatic oversight under Stiefel Program activities. DM and LC: development support for radiomics workflow and curation of the radiomics-based image features. AM: supervision of image segmentation/image post-processing and clinical data collection, and manuscript editing. SL and CF: co-corresponding authors, primary investigators, conceived, coordinated, and directed all study activities, responsible for data collection, project integrity, manuscript content and editorial oversight and correspondence, direct oversight of trainee personnel. AR: co-corresponding author, primary investigator, conceived, coordinated, and directed all study activities, supervised SB: toward building the FPCA and random forest models, responsible for project integrity, manuscript content, editorial oversight and correspondence, analytic support, and conceptual advice regarding model construction. Preliminary analyses and portions of this data were presented as a poster at the 2018 American Society of Radiation Oncology (ASTRO) Multidisciplinary Head and Neck Cancer Symposium, February 15–17, 2018, Scottsdale, AZ, USA.

## Conflict of Interest

The authors declare that the research was conducted in the absence of any commercial or financial relationships that could be construed as a potential conflict of interest.

## Supplementary Material

Supplementary Figure 1 ROC curve for the FPCA-based temporal trajectory model with optimal operating point. The optimal operating point of the ROC curve is identified by maximizing the Youden's index. The corresponding sensitivity and specificity values for the optimal operating point are shown.

Supplementary Figure 2 Confusion matrix for the ORN prediction task at the optimal operating point. The confusion matrix showing the classification performance by the FPCA-based temporal trajectory model at the optimal operating point of the ROC curve.

Supplementary Table 1 Computed tomography- derived intensity histogram, shape and texture analysis features set.

Supplementary Table 2 Filtered radiomic feature set. The final set of 16 features chosen after correlation filtering, which are used for building baseline, delta, and FPCA models.

Appendix A1 Step-by-step implementation guide for the model construction pipeline.

## References

[B1] Allison R. R. Patel R. M. McLawhorn R. A. (2014). Radiation oncology: physics advances that minimize morbidity. Fut Oncol. 10, 2329–2344. 10.2217/fon.14.176 25525843

[B2] Allison R. R. Sibata C. Patel R. (2013). Future radiation therapy: photons, protons and particles. Fut Oncol. 9, 493–504. 10.2217/fon.13.13 23560373

[B3] Amadasun M. King R. (1989). Textural features corresponding to textural properties. IEEE Transact. Syst. Man Cybernet. 19, 1264–1274. 10.1109/21.44046

[B4] Ang K. K. Harris J. Wheeler R. Weber R. Rosenthal D. I. Nguyen-Tân P. F.. (2010). Human papillomavirus and survival of patients with oropharyngeal cancer. N. Engl. J. Med. 363, 24–35. 10.1056/NEJMoa0912217 20530316 PMC2943767

[B5] Aue A. Norinho D. D. Hörmann S. (2015). On the prediction of stationary functional time series. J Am Statist Associat. 110, 378–392. 10.1080/01621459.2014.909317

[B6] Barua S. Elhalawani H. Volpe S. Feghali K. A. Yang P. Ng S. P.. (2020). Discovering early imaging biomarkers of osteoradionecrosis in oropharyngeal cancer by characterization of temporal changes in computed tomography mandibular radiomic features. medRxiv. 2020:2020.10.09.20208827. 10.1101/2020.10.09.20208827

[B7] Barua S. Solis L. Parra E. R. Uraoka N. Jiang M. Wang H.. (2018). A functional spatial analysis platform for discovery of immunological interactions predictive of low-grade to high-grade transition of pancreatic intraductal papillary mucinous neoplasms. Cancer Informat. 17:1176935118782880. 10.1177/1176935118782880 30013304 PMC6043922

[B8] Ben-David M. A. Diamante M. Radawski J. D. Vineberg K. A. Stroup C. Murdoch-Kinch C. A.. (2007). Lack of osteoradionecrosis of the mandible after intensity-modulated radiotherapy for head and neck cancer: likely contributions of both dental care and improved dose distributions. Int J Radiation Oncol Biol Phys. 68, 396–402. 10.1016/j.ijrobp.2006.11.059 17321069 PMC2702207

[B9] Berrendero J. R. Justel A. Svarc M. (2011). Principal components for multivariate functional data. Computat. Statist. Data Analysis. 55, 2619–2634. 10.1016/j.csda.2011.03.011

[B10] Block A. M. Cozzi F. Patel R. Surucu M. Hurst N. Jr.. (2018). Radiomics in head and neck radiation therapy: impact of metal artifact reduction. Int. J. Radiation Oncol. Biol. Phys. 99:E640. 10.1016/j.ijrobp.2017.06.2146

[B11] Bonner J. A. Harari P. M. Giralt J. Azarnia N. Shin D. M. Cohen R. B.. (2006). Radiotherapy plus cetuximab for squamous-cell carcinoma of the head and neck. N. Engl. J. Med. 354, 567–578. 10.1056/NEJMoa053422 16467544

[B12] Breiman L. (2001). Random forests. Machine Learn. 45, 5–32. 10.1023/A:1010933404324

[B13] Cacicedo J. Navarro A. Del Hoyo O. Gomez-Iturriaga A. Alongi F. Medina J. A.. (2016). Role of fluorine-18 fluorodeoxyglucose PET/CT in head and neck oncology: the point of view of the radiation oncologist. Br J Radiol. 89:20160217. 10.1259/bjr.20160217 27416996 PMC5124833

[B14] Cardot H. Faivre R. Goulard M. (2003). Functional approaches for predicting land use with the temporal evolution of coarse resolution remote sensing data. J. Appl. Statistics 30, 1185–1199. 10.1080/0266476032000107187

[B15] Chaturvedi A. K. Engels E. A. Pfeiffer R. M. Hernandez B. Y. Xiao W. Kim E.. (2011). Human papillomavirus and rising oropharyngeal cancer incidence in the United States. J. Clin. Oncol. 29, 4294–4301. 10.1200/JCO.2011.36.4596 21969503 PMC3221528

[B16] Chiou J.-M. Chen Y.-T. Yang Y.-F. (2014). Multivariate functional principal component analysis: a normalization approach. Statistica Sinica 24, 1571–1596. 10.5705/ss.2013.305

[B17] ClinicalTrials.gov S. (2020). ClinicalTrials.gov [Internet] Bethesda (MD): National Library of Medicine (US). 2000 Feb 29 -. Identifier NCT03145077, Dynamic Contrast-Enhanced Magnetic Resonance Imaging in Diagnosing Osteoradionecrosis in Patients With Head and Neck Cancer That Is Primary, Has Come Back, or Has Spread to Other Places in the Body (2020). Available online at: https://clinicaltrials.gov/ct2/show/NCT03145077?term=stephen+laianddraw=2andrank=1 (accessed Feb 21, 2021).

[B18] Dauxois J. Pousse A. Romain Y. (1982). Asymptotic theory for the principal component analysis of a vector random function: some applications to statistical inference. J. Multivariate Analysis 12, 136–154. 10.1016/0047-259X(82)90088-4

[B20] Elhalawani H. Kanwar A. Mohamed A. S. R. White A. Zafereo J. Wong A.. (2018). Investigation of radiomic signatures for local recurrence using primary tumor texture analysis in oropharyngeal head and neck cancer patients. Sci. Rep. 8:1524. 10.1038/s41598-017-14687-0 29367653 PMC5784146

[B21] Elhalawani H. Mohamed A. S. R. Kanwar A. Dursteler A. Rock C. D. Eraj S. E.. (2018b). EP-2121: serial parotid gland radiomic-based model predicts post-radiation xerostomia in oropharyngeal cancer. Radiother. Oncol. 127, S1167–S8. 10.1016/S0167-8140(18)32430-7

[B22] Elhalawani H. E. Mohamed A. S. R. Volpe S. Yang P. Campbell S. Granberry R.. (2018a). PO-0991: serial tumor radiomic features predict response of head and neck cancer treated with Radiotherapy. Radiother. Oncol. 127:S551. 10.1016/S0167-8140(18)31301-X

[B23] Fan H. Kim S. M. Cho Y. J. Eo M. Y. Lee S. K. Woo K. M. (2014). New approach for the treatment of osteoradionecrosis with pentoxifylline and tocopherol. Biomater. Res. 18:13. 10.1186/2055-7124-18-13 26331064 PMC4552457

[B24] Foutz N. Z. Jank W. (2010). Research note-prerelease demand forecasting for motion pictures using functional shape analysis of virtual stock markets. Market. Sci. 29, 568–579. 10.1287/mksc.1090.0542

[B25] Freymann J. B. Kirby J. S. Perry J. H. Clunie D. A. Jaffe C. C. (2012). Image data sharing for biomedical research—meeting HIPAA requirements for de-identification. J. Digital Imaging 25, 14–24. 10.1007/s10278-011-9422-x 22038512 PMC3264712

[B26] Garden A. S. Dong L. Morrison W. H. Stugis E. M. Glisson B. S. Frank S. J.. (2013). Patterns of disease recurrence following treatment of oropharyngeal cancer with intensity modulated radiation therapy. Int. J. Radiat. Oncol. Biol. Phys. 85, 941–947. 10.1016/j.ijrobp.2012.08.004 22975604

[B27] Garg A. Tai K. (2013). Comparison of statistical and machine learning methods in modelling of data with multicollinearity. Int. J. Modell. Identificat. Control 18, 295–312. 10.1504/IJMIC.2013.053535

[B28] Gomez D. R. Estilo C. L. Wolden S. L. Zelefsky M. J. Kraus D. H. Wong R. J.. (2011). Correlation of osteoradionecrosis and dental events with dosimetric parameters in intensity-modulated radiation therapy for head-and-neck cancer. Int. J. Radiat. Oncol. Biol. Phys. 81:e207–e13. 10.1016/j.ijrobp.2011.02.003 21570202

[B29] Hamilton J. D. Lai S. Y. Ginsberg L. E. (2012). Superimposed infection in mandibular osteoradionecrosis: diagnosis and outcomes. J. Computer Assisted Tomogr. 36, 725–731. 10.1097/RCT.0b013e3182702f09 23192211 PMC3917313

[B30] Happ C. Greven S. (2018). Multivariate functional principal component analysis for data observed on different (Dimensional) domains. J. Am. Statistical Associat. 113, 649–659. 10.1080/01621459.2016.1273115

[B31] Haralick R. M. Shanmugam K. Dinstein I. (1973). Textural features for image classification. IEEE Transact. Syst. Man Cybernet. SMC-3, 610-21. 10.1109/TSMC.1973.4309314

[B32] Jacobson A. S. Buchbinder D. Hu K. Urken M. L. (2010). Paradigm shifts in the management of osteoradionecrosis of the mandible. Oral Oncol. 46, 795–801. 10.1016/j.oraloncology.2010.08.007 20843728

[B33] Joint H. Neck Radiotherapy M. R. (2016). Dynamic contrast-enhanced MRI detects acute radiotherapy-induced alterations in mandibular microvasculature: prospective assessment of imaging biomarkers of normal tissue injury. Sci. Rep. 6:29864. 10.1038/srep29864 27499209 PMC4976364

[B34] Kuhn M. (2008). Building predictive models in R using the caret *Package*. 2008. 28,26. 10.18637/jss.v028.i05

[B35] Landberg T. Chavaudra J. Dobbs J. Gerard J. P. Hanks G. Horiot J. C.. (1999). Report 62. J. Int. Commission Radiat. Units Measurements os32, NP-NP. 10.1093/jicru/os32.1.Report62

[B37] Leijenaar R. T. H. Carvalho S. Hoebers F. J. P. Aerts H. J. W. L. van Elmpt W. J. C. Huang S. H.. (2015). External validation of a prognostic CT-based radiomic signature in oropharyngeal squamous cell carcinoma. Acta Oncolog. 54, 1423–1429. 10.3109/0284186X.2015.1061214 26264429

[B38] Liang C. Huang Y. He L. Chen X. Ma Z. Dong D.. (2016). The development and validation of a CT-based radiomics signature for the preoperative discrimination of stage I-II and stage III-IV colorectal cancer. Oncotarget. 7, 31401–31412. 10.18632/oncotarget.8919 27120787 PMC5058766

[B39] Liaw A. Wiener M. (2002). Classification and regression by randomForest. R News. 2, 18–22.

[B40] Mackin D. Fave X. Zhang L. Fried D. Yang J. Taylor B.. (2015). Measuring CT scanner variability of radiomics features. Invest. Radiol. 50, 757–765. 10.1097/RLI.0000000000000180 26115366 PMC4598251

[B41] Mackin D. Fave X. Zhang L. Yang J. Jones A. K. Ng C. S.. (2017). Harmonizing the pixel size in retrospective computed tomography radiomics studies. PLoS ONE 12:e0178524. 10.1371/journal.pone.0178524 28934225 PMC5608195

[B42] Mann H. B. Whitney D. R. (1947). On a test of whether one of two random variables is stochastically larger than the other. Ann Math Statist. 18, 50–60. 10.1214/aoms/1177730491

[B43] Marx R. E. (1983). Osteoradionecrosis: a new concept of its pathophysiology. J Oral Maxillofac Surg. 41:283–8. 10.1016/0278-2391(83)90294-X 6572704

[B43a] Materka A. Strzelecki M. (1998). Texture Analysis Methods —A Review. Institute of Electronics, Technical University of Lodz, Brussels.

[B44] Mendenhall W. M. (2004). Mandibular osteoradionecrosis. J. Clin. Oncol. 22, 4867–4868. 10.1200/JCO.2004.09.959 15520050

[B45] Mohamed A. S. R. Hobbs B. P. Hutcheson K. A. Murri M. S. Garg N. Song J.. (2017). Dose-volume correlates of mandibular osteoradionecrosis in Oropharynx cancer patients receiving intensity-modulated radiotherapy: results from a case-matched comparison. Radiother. Oncol. 124, 232–239. 10.1016/j.radonc.2017.06.026 28733053 PMC5572506

[B46] Nardone V. Tini P. Carbone S. F. Grassi A. Biondi M. Sebaste L.. (2017). Bone texture analysis using CT-simulation scans to individuate risk parameters for radiation-induced insufficiency fractures. Osteoporosis Int. 28, 1915–1923. 10.1007/s00198-017-3968-5 28243706

[B47] Oh H.-K. Chambers M. S. Garden A. S. Wong P.-F. Martin J. W. (2004). Risk of osteoradionecrosis after extraction of impacted third molars in irradiated head and neck cancer patients. J. Oral Maxillofacial Surg. 62, 139–144. 10.1016/j.joms.2003.08.009 14762744

[B48] Oh H.-K. Chambers M. S. Martin J. W. Lim H.-J. Park H.-J. (2009). Osteoradionecrosis of the mandible: treatment outcomes and factors influencing the progress of osteoradionecrosis. J. Oral and Maxillofacial Surg. 67, 1378–1386. 10.1016/j.joms.2009.02.008 19531406

[B49] Ollivier M. Le Corroller T. Blanc G. Parratte S. Champsaur P. Chabrand P.. (2013). Radiographic bone texture analysis is correlated with 3D microarchitecture in the femoral head, and improves the estimation of the femoral neck fracture risk when combined with bone mineral density. Eur. J. Radiol. 82, 1494–1498. 10.1016/j.ejrad.2013.04.042 23756323

[B50] Pan H. Y. Haffty B. G. Falit B. P. Buchholz T. A. Wilson L. D. Hahn S. M.. (2016). Supply and demand for radiation oncology in the United States: updated projections for 2015 to 2025. Int. J. Radiation Oncol. Biol. Phys. 96, 493–500. 10.1016/j.ijrobp.2016.02.064 27209499

[B51] Roberts M. G. Graham J. Devlin H. (2013). Image texture in dental panoramic radiographs as a potential biomarker of osteoporosis. IEEE Transact. Bio-Medical Eng. 60, 2384–2392. 10.1109/TBME.2013.2256908 23568478

[B52] Robin X. Turck N. Hainard A. Tiberti N. Lisacek F. Sanchez J. C.. (2011). pROC: an open-source package for R and S+ to analyze and compare ROC curves. BMC Bioinformatics 12:77. 10.1186/1471-2105-12-77 21414208 PMC3068975

[B53] Shafiq-ul-Hassan M. Zhang G. G. Latifi K. Ullah G. Hunt D. C. Balagurunathan Y.. (2017). Intrinsic dependencies of CT radiomic features on voxel size and number of gray levels. Med. Phys. 44, 1050–1062. 10.1002/mp.12123 28112418 PMC5462462

[B54] Shang H. L. (2014). A survey of functional principal component analysis. AStA Adv Stat Anal. 98, 121–142. 10.1007/s10182-013-0213-1

[B55] Shou H. Zipunnikov V. Crainiceanu C.M. Greven S. (2015). Structured functional principal component analysis. Biometrics 71, 247–257. 10.1111/biom.12236 25327216 PMC4383722

[B57] Tsai C. J. Hofstede T. M. Sturgis E. M. Garden A. S. Lindberg M. E. Wei Q.. (2013). Osteoradionecrosis and radiation dose to the mandible in patients with oropharyngeal cancer. Int. J. Radiat. Oncol. Biol. Phys. 85, 415–420. 10.1016/j.ijrobp.2012.05.032 22795804

[B58] Tsien C. Cao Y. Chenevert T. (2014). Clinical applications for diffusion magnetic resonance imaging in radiotherapy. Semin. Radiat. Oncol. 24, 218–226. 10.1016/j.semradonc.2014.02.004 24931097 PMC4170230

[B59] Tucker J. R. Xu L. Sturgis E. M. Mohamed A. S. R. Hofstede T. M. Chambers M. S.. (2016). Osteoradionecrosis in patients with salivary gland malignancies. Oral Oncol. 57, 1–5. 10.1016/j.oraloncology.2016.03.006 27208837 PMC4990067

[B60] Unser M. (1986). Sum and difference histograms for texture classification. IEEE Transact. Pattern Anal. Machine Intelligence PAMI-8, 118–125. 10.1109/TPAMI.1986.4767760 21869331

[B61] Wong A. J. Kanwar A. Mohamed A. S. Fuller C. D. (2016). Radiomics in head and neck cancer: from exploration to application. Translat. Cancer Res. 5, 371–382. 10.21037/tcr.2016.07.18 30627523 PMC6322843

[B62] Wong A. T. T. Lai S. Y. Gunn G. B. Beadle B. M. Fuller C. D. Barrow M. P.. (2017). Symptom burden and dysphagia associated with osteoradionecrosis in long-term oropharynx cancer survivors: a cohort analysis. Oral Oncol. 66, 75–80. 10.1016/j.oraloncology.2017.01.006 28249651 PMC5336132

[B63] Yang P. Mackin D. Chen C. Mohamed A. S. R. Elhalawani H. Shi Y.. (2018). Discrimination of epstein-barr virus status in NPC Using CT-derived radiomics features: linking imaging phenotypes to tumor biology. Int. J. Radiat. Oncol. Biol. Phys. 100:1361. 10.1016/j.ijrobp.2017.12.142

[B64] Yang X. Tridandapani S. Beitler J. J. Yu D. S. Yoshida E. J. Curran W. J.. (2012). Ultrasound GLCM texture analysis of radiation-induced parotid-gland injury in head-and-neck cancer radiotherapy: an *in vivo* study of late toxicity. Med. Phys. 39, 5732–5739. 10.1118/1.4747526 22957638 PMC3443195

[B65] Yin Q. Hung S.-C. Wang L. Lin W. Fielding J. R. Rathmell W. K.. (2017). Associations between tumor vascularity, vascular endothelial growth factor expression and PET/MRI radiomic signatures in primary clear-cell–renal-cell-carcinoma: proof-of-concept study. Sci. Rep. 7:43356. 10.1038/srep43356 28256615 PMC5335708

[B66] Youden W. J. (1950). Index for rating diagnostic tests. Cancer. 3, 32–35. 10.1002/1097-0142(1950)3:1andlt;32::AID-CNCR2820030106andgt;3.0.CO;2-3 15405679

[B67] Zar J. H. (2005). Spearman rank correlation, in Encyclopedia of Biostatistics, eds P. Armitage and T. Colton. 10.1002/0470011815.b2a15150

[B68] Zhang L. Fried D. V. Fave X. J. Hunter L. A. Yang J. Court L. E. (2015). IBEX: an open infrastructure software platform to facilitate collaborative work in radiomics. Med. Phys. 42, 1341–1353. 10.1118/1.4908210 25735289 PMC5148126

[B69] Zhang W. Zhang X. Yang P. Blanchard P. Garden A. S. Gunn B.. (2017). Intensity-modulated proton therapy and osteoradionecrosis in oropharyngeal cancer. Radiother. Oncol. 123, 401–405. 10.1016/j.radonc.2017.05.006 28549794 PMC5779856

