# Supplementary figures and images for: Computed Tomography Radiomics Kinetics as Early Imaging Correlates of Osteoradionecrosis in Oropharyngeal Cancer Patients
Source: Front Artif Intell. 2021 Apr 9;4:618469. doi: 10.3389/frai.2021.618469 (PMC8063205; doi:10.3389/frai.2021.618469)

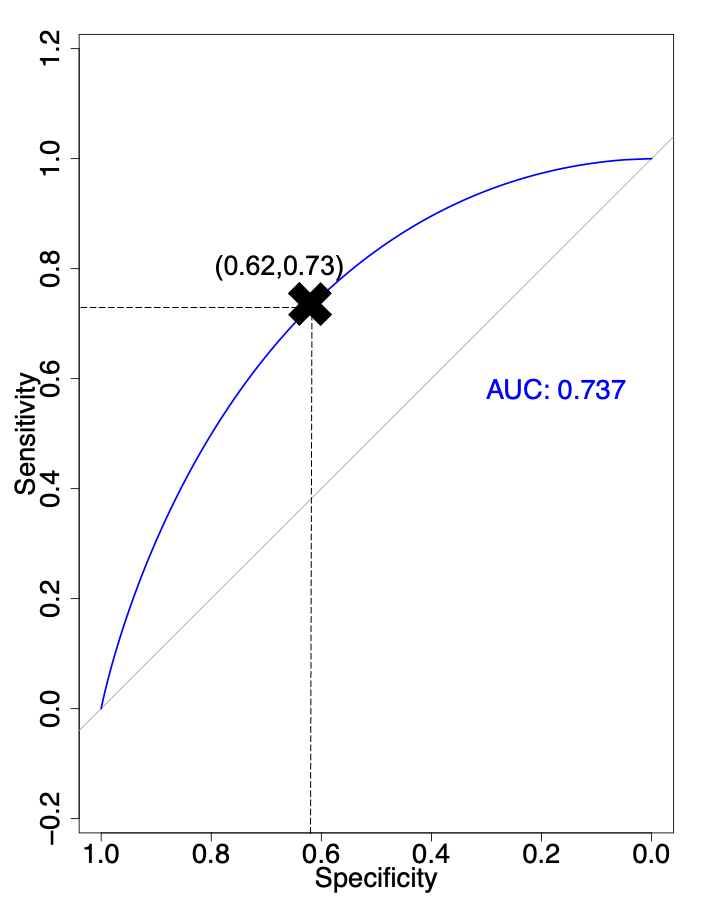

Supplement: Supplementary Figure 1 — ROC curve for the FPCA-based temporal trajectory model with optimal operating point. The optimal operating point of the ROC curve is identified by maximizing the Youden's index. The corresponding sensitivity and specificity values for the optimal operating point are shown. [file Image_1.TIFF]

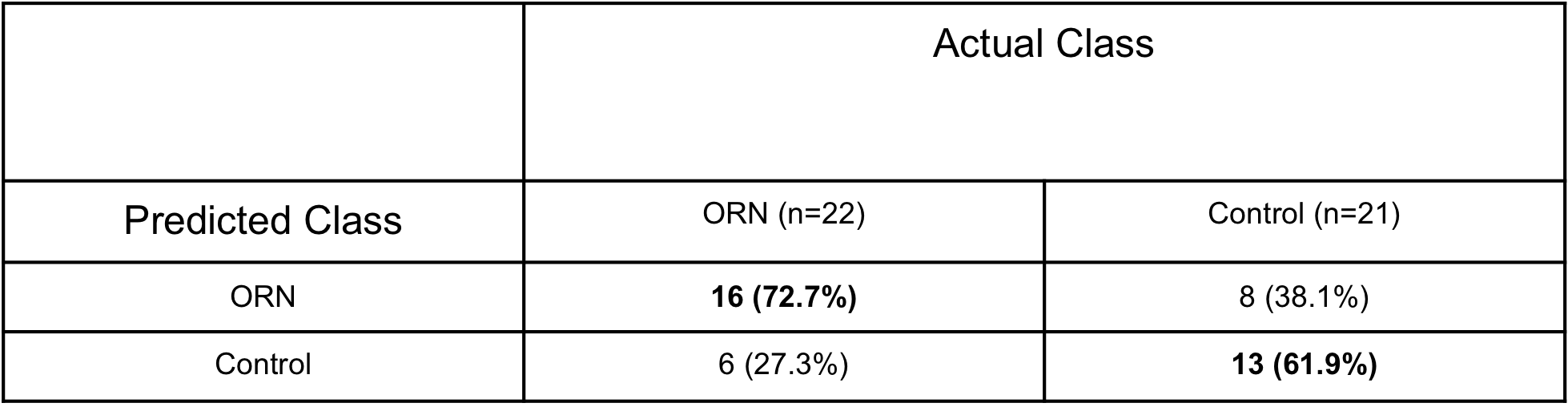

Supplement: Supplementary Figure 2 — Confusion matrix for the ORN prediction task at the optimal operating point. The confusion matrix showing the classification performance by the FPCA-based temporal trajectory model at the optimal operating point of the ROC curve. [file Image_2.TIFF]
