# Supplementary material for: Computed Tomography Radiomics Kinetics as Early Imaging Correlates of Osteoradionecrosis in Oropharyngeal Cancer Patients
Source: Front Artif Intell. 2021 Apr 9;4:618469. doi: 10.3389/frai.2021.618469 (PMC8063205; doi:10.3389/frai.2021.618469)
Supplement: Supplementary Table 1 — Computed tomography- derived intensity histogram, shape and texture analysis features set. [file Table_1.DOCX]

**S1 Table. Computed tomography- derived intensity histogram, shape and texture analysis features set**

| **Feature Category** | **Features** |
| --- | --- |
| Gray Level Cooccurrence Matrix 25  Gray Level Cooccurrence Matrix 3 | Auto-Correlation |
|  | Cluster Prominence |
|  | Cluster Shade |
|  | Cluster Tendency |
|  | Contrast |
|  | Correlation |
|  | Difference Entropy |
|  | Dissimilarity |
|  | Energy |
|  | Entropy |
|  | Homogeneity |
|  | Homogeneity 2 |
|  | Information Measure Correlation 1 |
|  | Information Measure Correlation 2 |
|  | Inverse Diff Moment Norm |
|  | Inverse Diff Norm |

|  | Inverse Variance |
| --- | --- |
|  | Max Probability |
|  | Sum Average |
|  | Sum Entropy |
|  | Sum Variance |
|  | Variance |
| GrayLevelRunLengthMatrix25 | Gray Level Non- uniformity |
|  | High Gray Level Run Emphasis |
|  | Long Run Emphasis |
|  | Long Run High Gray Level Emphasis |
|  | Long Run Low Gray Level Emphasis |
|  | Low Gray Level Run Emphasis |
|  | Run Length Non- uniformity |
|  | Run Percentage |
|  | Short Run Emphasis |
|  | Short Run High Gray Level Emphasis |
|  | Short Run Low Gray Level Emphasis |

| Neighbor Intensity Difference 25  Neighbor Intensity Difference 3 | Busyness |
| --- | --- |
|  | Coarseness |
|  | Complexity |
|  | Contrast |
|  | Texture Strength |
| Intensity Direct | Energy |
|  | Global Entropy |
|  | Global Max |
|  | Global Mean |
|  | Global Median |
|  | Global Min |
|  | Global Std |
|  | Global Uniformity |
|  | Inter-Quartile Range |
|  | Kurtosis |
|  | Local Entropy Max |
|  | Local Entropy Mean |
|  | Local Entropy Median |
|  | Local Entropy Min |
|  | Local Entropy Std |

|  | Local Range Max |
| --- | --- |
|  | Local Range Mean |
|  | Local Range Median |
|  | Local Range Min |
|  | Local Range Std |
|  | Local Std Max |
|  | Local Std Mean |
|  | Local Std Median |
|  | Local Std Min |
|  | Local Std Std |
|  | Mean Absolute Deviation |
|  | Median Absolute Deviation |
|  | Percentile |

|  | Quantile |
| --- | --- |
|  | Range |
|  | Root Mean Square |
|  | Skewness |
|  | Variance |
| Intensity Histogram | Inter-Quartile Range |
|  | Kurtosis |
|  | Mean Absolute Deviation |
|  | Median Absolute Deviation |
|  | Percentile |
|  | Percentile Area |
|  | Quantile |
|  | Range |
|  | Skewness |
| Shape | Compactness 1 |
|  | Compactness 2 |
|  | Convex |
|  | Convex Hull Volume |
|  | Convex Hull Volume 3D |
|  | Mass |
|  | Max 3D Diameter |

|  | Mean Breadth |
| --- | --- |
|  | Number Of Voxel |
|  | Orientation |
|  | Roundness |
|  | Spherical Disproportion |
|  | Sphericity |
|  | Surface Area |
|  | Surface Area Density |
|  | Volume |
