# Supplementary material for: Computed Tomography Radiomics Kinetics as Early Imaging Correlates of Osteoradionecrosis in Oropharyngeal Cancer Patients
Source: Front Artif Intell. 2021 Apr 9;4:618469. doi: 10.3389/frai.2021.618469 (PMC8063205; doi:10.3389/frai.2021.618469)
Supplement: Appendix A1 — Step-by-step implementation guide for the model construction pipeline. [file Data_Sheet_1.docx]

**A1 Appendix. A step-by-step implementation guide for the model construction pipeline**

Step 1: Correlation filtering

We compute a pair-wise Spearman correlation for all features. We then use the ‘findCorrelation’ function in the R package **caret** to find a set of 16 features with the correlation between them being lower than a pre-defined threshold (0.5). We ensure that the volume and mean intensity features are preserved in the filtering process. Step 1 thus consists of:

(a) Find features that have correlation with volume lower than the defined threshold.

(b) Find features that have correlation with intensity lower than the defined threshold.
(c) Find the common features in (a) and (b).

(d) Find a subset of features that have correlation with each other lower than the defined threshold.

Step 2: Baseline and delta model construction

(a) Baseline: Use only the features at baseline

(b) Delta (2-month follow-up): Computed as f_delta (2-months)_ = (f_post-RT2_– f_baseline_)/ f_baseline_

(c) Delta (6-month follow-up): Computed as f_delta (6-months)_ = (f_post-RT6_– f_baseline_)/ f_baseline_

Step 3: Temporal trajectory model construction using MFPCA

(a) Timepoint interpolation: Since the time points are not uniformly spaced, we used cubic spline sequence completion (**spline** function in MATLAB) to fill in the values of radiomic features at intermediate monthly time points. Thus, we get a total of 8 timepoints, where timepoints 1 (pre-IMRT), 4 (post-RT2),

and 8 (post-RT6) are known a-priori, while timepoints 2 (end of RT),3 (post-RT1),5 (post-RT3),6 (post-RT4), and 7 (post-RT5) are interpolated.

(b) MFPCA analysis: We used the ‘MFPCA’ function in the **MFPCA** package to transform the 10 radiomic features into a set of 8 MFPCA features.

Step 4: Random Forest (RF) analysis

(a) Building the RF model: The RF model was built using the ‘randomForest’ function in the **randomForest** package in R on each model- Baseline, Delta (2-month follow-up), Delta (2-month follow-up), and Temporal trajectory using MFPCA. The number of trees used was 500.

(b) Constructing ROC curves: The ‘ROC’ function in the **pROC** package was used to generate the ROC curves for each model. The ‘smooth’ function from the same package was used to smooth the raw ROC curves using binormal smoothing and generate visually intelligible ROC curves as shown in the paper.

(c) Finding optimal operating point: The optimal operating point on the ROC curve is computed by maximizing the Youden index (max(sensitivity+specificity-1)) across all points on the ROC curve.
